# Bilateral Fundus Excyclotorsion in Unilateral Superior Oblique Palsy Confirmed by MR Imaging

**DOI:** 10.3390/jcm9061829

**Published:** 2020-06-11

**Authors:** Eun Hee Hong, Hee Kyung Yang, Jae Hyoung Kim, Jeong-Min Hwang

**Affiliations:** 1 Department of Ophthalmology, Hanyang University College of Medicine, Hanyang University Guri Hospital, 153, Gyeongchun-ro, Guri-si, Gyeonggi-do 11923, Korea; prayseven@gmail.com; 2 Departments of Ophthalmology, Seoul National University College of Medicine, Seoul National University Bundang Hospital, 82 Gumi-ro 173beon-gil, Bundang-gu, Seongnam-si, Gyeonggi-do 13620, Korea; nan282@snu.ac.kr; 3 Departments of Radiology, Seoul National University College of Medicine, Seoul National University Bundang Hospital, 82 Gumi-ro 173beon-gil, Bundang-gu, Seongnam-si, Gyeonggi-do 13620, Korea

**Keywords:** unilateral superior oblique palsy, superior oblique hypoplasia, bilateral superior oblique palsy, fundus excyclotorsion

## Abstract

Purpose: To determine whether bilateral fundus excyclotorsion is helpful in distinguishing bilateral superior oblique palsy (SOP) from unilateral SOP by investigating bilateral fundus excyclotorsion in unilateral SOP and comparing the features with bilateral SOP using fundus photographs. Methods: This retrospective cohort study included a total of 212 subjects who were diagnosed with unilateral SOP with hypoplasia of a single superior oblique (SO) muscle and 7 subjects with clinically diagnosed bilateral SOP. Fundus excyclotorsion measured by modified fovea–disc angles and inter-eye differences in cyclotorsion angles (the difference in fundus excyclotorsion angles: paretic eye or hypertropic eye in primary gaze–fellow eye), and subjective cyclotorsion were compared between groups of unilateral SOP with bilateral fundus excyclotorsion (SOP_BE_) and bilateral SOP. Results: Bilateral fundus excyclotorsion was found in 18 out of 212 patients (8.5%) in the unilateral SOP group, and 7 out of 7 patients (100%) in the bilateral SOP group. Among the 25 patients with bilateral fundus excyclotorsion, the mean angle of excyclotorsion (5.7° ± 4.7° vs. 7.6° ± 4.3°, *p* = 0.125) and the inter-eye differences (0.7° ± 3.6° vs. 0.5° ± 5.8°, *p* = 0.615) were not significantly different between the unilateral SOP_BE_ and bilateral SOP groups. The degree of subjective excyclotorsion was significantly larger in the bilateral SOP group compared with the unilateral SOP_BE_ group (16.0 ± 5.5 vs. 4.6 ± 4.3, *p* = 0.002). Conclusion: Bilateral fundus excyclotorsion was demonstrated not only in bilateral SOP, but also in unilateral SOP at a rate of 8.5%. Bilateral fundus excyclotorsion alone did not prove to be a specific sign in distinguishing bilateral SOP from unilateral SOP.

## 1. Introduction

Superior oblique palsy (SOP) is the most common paralytic strabismus which causes cyclotorsion [1,2]. When diagnosing SOP, bilateral SOP needs to be distinguished from unilateral SOP, as it is essential to establish the surgical plan [3]. The term “masked” bilateral SOP has been used to describe the development of an apparent SOP in the contralateral eye in which the SOP initially appeared to be unilateral before surgical correction, and it emphasizes the importance of this discrimination [3]. On the other hand, surgical overcorrection of unilateral SOP can cause consecutive contralateral SOP that exactly mimics masked bilateral SOP [4]. Thus, many studies have tried to reveal sensitive signs to distinguish bilateral SOP from unilateral SOP preoperatively [2,5].

Bilateral fundus excyclotorsion, as one of those sensitive signs, is usually regarded as a distinct clinical feature of bilateral SOP to detect masked bilateral SOP in patients with presumed unilateral SOP [5,6]. With the advance in imaging modalities, recent investigations using magnetic resonance imaging (MRI) to confirm a hypoplastic superior oblique muscle (SO) suggested that clinical tests including the Parks three-step test, previously described as the most reliable test in diagnosing SOP, were not of high diagnostic value [7,8]. Moreover, Muthusamy et al. reported the poor sensitivity of the Bielschowsky head-tilt test, the Parks three-step test, and reversal of the hypertropia in distinguishing bilateral SOP from unilateral SOP [5]. However, the diagnostic value of bilateral fundus excyclotorsion in bilateral SOP has not been validated. Meanwhile, in unilateral SOP, ipsilateral ocular excyclotorsion is one of the well-known characteristic clinical features [2,9]. On the other hand, unilateral fundus excyclotorsion in the non-paretic eye has been reported in 25% to 38% of unilateral SOP [10,11,12,13]. However, whether bilateral fundus excyclotorsion is a unique feature of bilateral SOP and is not found in unilateral SOP has not been thoroughly investigated.

In this study, we determined the incidence and patterns of bilateral fundus excyclotorsion in unilateral SOP patients with hypoplasia of the ipsilateral SO to find out if bilateral fundus excyclotorsion is truly a critical sign in distinguishing between bilateral and unilateral SOP.

## 2. Materials and Methods

This retrospective study was approved by the Institutional Review Board of Seoul National University Bundang Hospital (B-1511-322-102), and all research conducted adhered to the tenets of the Declaration of Helsinki. Informed consent was not given, as subject records and information were anonymized and de-identified prior to analysis.

### 2.1. Subjects

Subjects who were diagnosed with unilateral or bilateral SOP and underwent fundus photography with internal fixators at Seoul National University Bundang Hospital between January 2012 and February 2018 were reviewed. The results of high-resolution thin-section magnetic resonance imaging (MRI), performed for the evaluation of the trochlear nerve and superior oblique muscle (SO), were also reviewed. Diagnosis of unilateral SOP was made by the following criteria: (1) any hyperdeviation in primary gaze position; (2) unilateral underdepression in adduction with or without overelevation in adduction; (3) the absence of reversal of hypertropia on ipsilateral side gaze and contralateral head tilt; and (4) lack of evidence of other ocular motility disorders causing vertical deviation, including contracture of the vertical rectus muscles, except for secondary contracture of the superior rectus (SR) muscle associated with SOP [14,15,16], paresis of vertical muscles, previous muscle surgery, skew deviation, myasthenia gravis, and vertical deviations associated with horizontal strabismus [17], as well as structural abnormalities such as craniosynostosis or muscle pulley heterotopy [18,19] without evidence of SO hypoplasia on MRI. Patients diagnosed with unilateral SOP were included in this study only when confirmed by MRI to have unilateral SO hypoplasia (unilateral SOP group). The clinical diagnostic criteria of bilateral SOP were as follows: (1) bilateral SO muscle dysfunction; underdepression in adduction with or without overelevation in adduction; (2) reversing hypertropia on side gazes or in the inferior tertiary positions of gaze; (3) any V-patterns; and (4) positive Bielschowsky head tilt tests to both sides [2,3]. Patients diagnosed with bilateral SOP were included in this study if they were confirmed by MRI to have bilateral SO hypoplasia or had a clear history of head trauma preceding the onset of signs and symptoms (bilateral SOP group). Medical records of ophthalmic examinations, including prism and alternate cover measurements and an ocular motility assessment for the oblique muscles, and medical history were reviewed in all subjects. Age at onset of sign/symptoms, symptom duration, etiology (congenital or acquired), laterality of the paretic eye, and presence of head tilt were recorded. Subjective cyclotorsion in primary gaze measured with the double Maddox rod test was also recorded, if performed. Subjective cyclotorsion angles were recorded as a positive degree for excyclotorsion, and a negative degree for incyclotorsion.

### 2.2. Measurement of Fundus Cyclotorsion

Fundus cyclotorsion was evaluated by fundus photographs obtained with KOWA VX-10 (Kowa Company, Ltd., Tokyo, Japan) and TRC-50IA (Topcon, Inc., Tokyo, Japan) fundus cameras using internal fixation through a built-in internal fixator, while the fellow eye was not occluded. The fundus photographs of all subjects in the unilateral SOP and bilateral SOP groups were reviewed. If the fovea was located below the horizontal bottom line of the optic disc, the eye was classified as excyclotorsion; if the fovea was above the horizontal line passing the lower third of the optic disc, the eye was classified as incyclotorsion [20,21]. Accordingly, patients in the unilateral SOP group who presented with bilateral fundus excyclotorsion on fundus photographs were classified as the unilateral SOP with bilateral excyclotorsion (unilateral SOP_BE_) group.

In addition to this qualitative evaluation of fundus cyclotorsion, we also measured fundus cyclotorsion quantitatively in the unilateral SOP_BE_ and bilateral SOP groups. Based on the methods described by Kushner and Hariharan [9], the number of degrees of foveal rotation from the horizontal bottom line of the optic disc were recorded. This measurement was used in our study because we thought that measuring this rotation angle is simpler, easily applicable, and less affected by inter-individual variation of the optic disc and fundus morphology than measuring the fovea-to-disc angle. The fundus cyclotorsion angle was measured twice and averaged by an investigator who was masked to the diagnosis using a semi-automated angle measurement tool embedded in the INFINITT PACS program. Fundus excyclotorsion angles were recorded as a positive value, and incyclotorsion angles were recorded as a negative value. We defined the “inter-eye excyclotorsion angle differences” as the difference in fundus excyclotorsion angles between the paretic eye and the fellow eye (paretic eye-fellow eye) for unilateral SOP and between the hypertropic eye in primary gaze and the fellow eye for bilateral SOP. The inter-eye difference of the fovea–to-disc angle in normal subjects was observed previously in a range of 0.0° to 4.4° based on fundus photographs by Bixenman and von Noorden [20], and more recently as a mean of 1.15° ± 1.39° by Jethani et al. [22]. The fovea-to-disc angle followed a normal distribution in a population-based study [23]. Therefore, we used a range covering approximately 95% of the data which falls within two standard deviations of the mean of normal subjects reported by Jethani et al. as a reference value (3.93°) for determining whether the fundus excyclotorsion was symmetric or asymmetric. An inter-eye excyclotorsion angle difference beyond this range was defined as “asymmetric bilateral excyclotorsion” and an inter-eye difference not exceeding this range was defined as “symmetric bilateral excyclotorsion”.

### 2.3. Magnetic Resonance Imaging: Trochlear Nerve and Superior Oblique Muscle

MRI was performed by using a 3T system (Intera Achieva; Philips Healthcare, Best, The Netherlands) with an 8-channel sensitivity encoding head coil, and general aspects of the MRI protocol were in accordance with our previous description [24]. Children younger than 6 years were sedated by chloral hydrate to minimize motion artifacts.

### 2.4. Main Outcome Measures 

Primarily, the proportion of subjects who presented with bilateral fundus excyclotorsion among subjects with unilateral SOP was calculated. Secondarily, fundus excyclotorsion angles in both eyes of each group, the inter-eye excyclotorsion angle differences, and subjective excyclotorsion angle in the unilateral SOP_BE_ and bilateral SOP groups were compared. Bilateral excyclotorsion was classified as asymmetric when the inter-eye excyclotorsion angle difference exceeded the range between −3.93° and 3.93°, otherwise, it was classified as symmetric. The correlation of binocular sensory status and the degree of excyclotorsion was investigated in the unilateral SOP_BE_ and bilateral SOP groups. 

### 2.5. Statistical Analyses

Statistical analyses were performed with the SPSS software version 20 (SPSS, Inc., Chicago, IL, USA). We compared groups using the Mann–Whitney U test and independent t-test for continuous variables, and Pearson’s Chi-square test for categorical data. A *p* value of <0.05 was considered statistically significant. 

## 3. Results

### 3.1. Subjects

Among the 212 patients who were diagnosed with unilateral SOP and showed ipsilateral hypoplasia of the SO muscle during the study period, 18 (8.5%) patients presented with bilateral fundus excyclotorsion and were classified as the unilateral SOP_BE_ group. Seven patients were diagnosed with bilateral SOP and were classified as the bilateral SOP group. Comparisons of clinical characteristics and fundus cyclotorsion between the unilateral SOP_BE_ group and bilateral SOP group were performed. All cases with unilateral SOP were congenital. Five out of seven from the bilateral SOP group underwent MRI. Two of them showed MRI findings of bilateral trochlear nerve agenesis and bilateral SO hypoplasia and were classified as congenital, while the other three showed normal MRI findings, but had a clear history of head trauma preceding the onset of signs and symptoms. Two patients from the bilateral SOP group who did not undergo MRI also had a clear history of head trauma preceding the onset of signs and symptoms. The clinical characteristics of subjects with unilateral SOP_BE_ and bilateral SOP are presented in Table 1. The mean age was older in the bilateral SOP group (*p* = 0.005, Table 1). Among the bilateral SOP group, the mean ages of the congenital and acquired subgroups were 50.0 ± 11.3 (42–58) and 48.8 ± 12.8 (34–65), respectively.

We confirmed that none of the patients in the unilateral SOP_BE_ group showed any suspicious feature of masked bilateral SOP [3]: a reversal of the hypertropia in any of the oblique fields of gaze, a “V” shift (difference in horizontal alignment between upgaze and downgaze greater than 20 PD), a small hypertropia in primary gaze with a large hypertropia of the same eye in adduction, a chin down rather than a head tilt posture, or a reversal of the hypertropia with head tilt to the contralateral side. Twelve patients in the unilateral SOP_BE_ group later underwent ipsilateral strabismus surgery, and none of them showed contralateral SOP postoperatively.

### 3.2. Bilateral Fundus Excyclotorsion in Unilateral and Bilateral SOP

Among the 18 subjects in the unilateral SOP_BE_ group, 12 (66.7%) subjects showed symmetric fundus excyclotorsion and 6 patients (33.3%) showed asymmetric fundus excyclotorsion. There were no significant differences in the ipsilateral and contralateral fundus excyclotorsion angles and subjective excyclotorsion between those with asymmetric excyclotorsion and symmetric excyclotorsion within the unilateral SOP_BE_ group (all *p* > 0.05).

**Figure 1:**
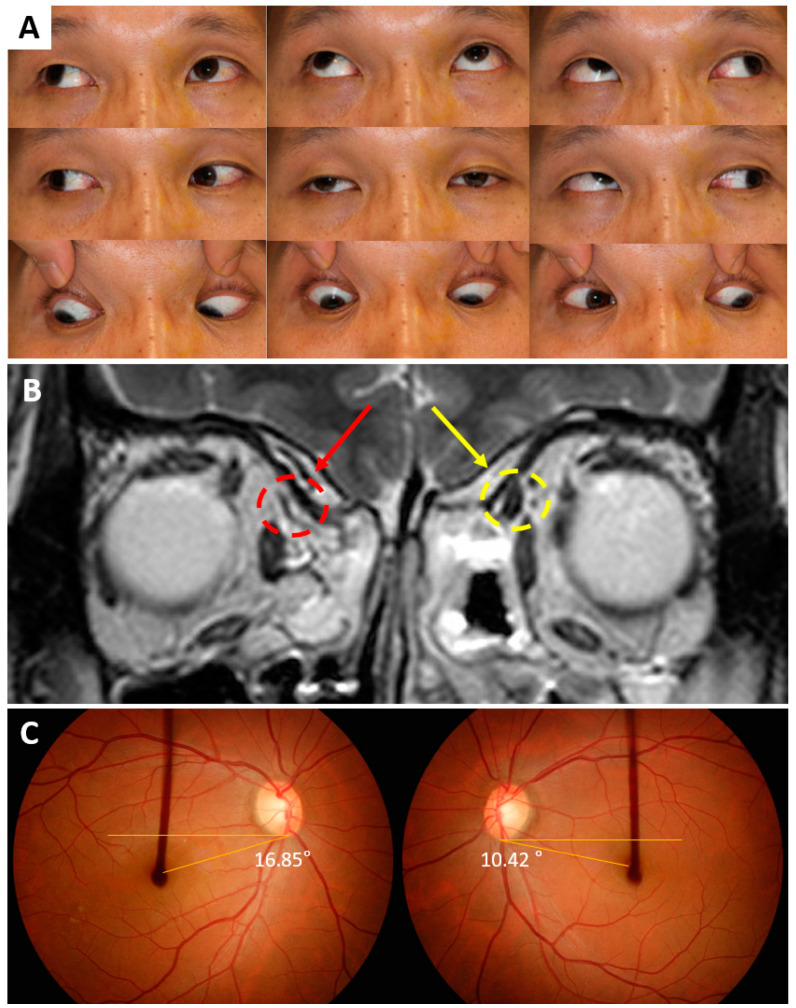
A representative case of a 45-year-old man with unilateral right superior oblique palsy and bilateral fundus excyclotorsion. (**A**) Ocular versions demonstrating increased elevation and decreased depression in adduction of the right eye. (**B**) The right superior oblique muscle is hypoplastic (red arrow) compared with the left (yellow arrow). (**C**) Bilateral fundus excyclotorsion on fundus photographs taken with an internal fixator. Single angle measurements are indicated in both eyes.

Figure 1 shows a representative case in the unilateral SOP_BE_ group of a 45-year-old man who had right SOP and bilateral fundus excyclotorsion. He had a misalignment of his right eye from childhood and showed right hypertropia and exotropia on the alternate prism and cover test in primary gaze position at distance and at near. The right eye showed overelevation and limited depression on adduction (Figure 1A) with no suspicious features of masked bilateral SOP. High-resolution MRI revealed unilateral SO hypoplasia (Figure 1B). Fundus photographs with an internal fixator featured 16.85 degrees of excyclotorsion in the right eye and 10.42 degrees of excyclotorsion in the left eye (Figure 1C). The average of two measurements of fundus excyclotorsion angles were 16.98 and 11.01 degrees in the right and left eyes, respectively.

Among the seven subjects with bilateral SOP, five (71.4%) subjects showed symmetric fundus excyclotorsion and two (28.6%) showed asymmetric bilateral fundus excyclotorsion.

In comparison of the unilateral SOP_BE_ group and bilateral SOP group, symptom duration was shorter (*p* = 0.001) and the degree of subjective excyclotorsion was worse in the bilateral SOP group than the unilateral SOP_BE_ group (*p* = 0.001). The fundus excyclotorsion angles in both eyes (paretic or hypertropic eye, *p* =0.125; fellow eye, *p* = 0.423) and the inter-eye excyclotorsion angle differences (*p* = 0.615) showed no significant differences between the unilateral SOP_BE_ and bilateral SOP groups.

There was no significant difference in the rate of asymmetric bilateral excyclotorsion between the two groups (22.2% in unilateral SOP_BE_ group vs. 28.6% in bilateral SOP group, *p* = 0.744).

### 3.3. Subjective Excyclotorsion in Unilateral SOP_BE_ vs. Bilateral SOP

Subjective excyclotorsion angles were recorded in 14 (77.8%) subjects in the unilateral SOP_BE_ group and 6 (85.7%) subjects in the bilateral SOP group. The angle of subjective excyclotorsion was significantly greater in the bilateral SOP group than the unilateral SOP_BE_ group (*p* = 0.002, Table 1).

## 4. Discussion

In this study, bilateral fundus excyclotorsion was present in 8.5% of unilateral SOP patients and most of them showed symmetric excyclotorsion between the paretic eye and non-paretic eye. The angle of excyclotorsion and inter-eye excyclotorsion angle differences were not significantly different between the unilateral SOP group with bilateral excyclotorsion and the bilateral SOP group. Therefore, bilateral fundus excyclotorsion may not be regarded as a specific sign for bilateral SOP. Meanwhile, the amount of subjective excyclotorsion was greater in patients with bilateral SOP compared with unilateral SOP with bilateral fundus excyclotorsion.

When evaluating fundus excyclotorsion, the horizontal bottom line of the optic disc has been used as a practical reference line for determining whether it is pathologic [25]. However, as Bixenman and von Noorden described, it is difficult to define an absolute cut-off value for pathologic fundus cyclotorsion due to the variations in normal eyes [20]. The fovea–disc angle is often used for the quantitative measurement of fundus cyclotorsion, but it has the limitations of an indeterminate cut-off value and inter-individual variations in the optic disc and fundus morphology. As Kushner et al. have mentioned, a normal torsional range of approximately 9 degrees above the bottom of the optic disc can be considered in most eyes [9]. Accordingly, we measured the fundus excyclotorsion angle as the rotation angle of the fovea below the horizontal bottom line of the optic disc in this study. By using the horizontal bottom line of the optic disc as the reference line for both the qualitative and quantitative measurements, we could measure fundus excyclotorsion more practically and simply, less affected by inter-individual variations.

In unilateral SOP, fundus excyclotorsion in the non-paretic eye has been reported in previous studies [10,11,12,13]. Kim et al. reported that patients with unilateral SOP showed a significantly larger disc–fovea angle in both eyes compared with controls, however, the “qualitative” fundus excyclotorsion was unilateral or absent in those patients [26]. Meanwhile, bilateral fundus excyclotorsion has been considered as an important sign for the diagnosis of bilateral SOP [3,6]. To our knowledge, no study has demonstrated bilateral fundus excyclotorsion in unilateral SOP. Kushner et al. previously observed that fundus excyclotorsion was switched from the paretic eye to the non-paretic eye in unilateral SOP patients and assumed that objective excyclotorsion that originally existed in the paretic eye may shift fully or “partially” to the non-paretic eye over time [9]. Accordingly, they noted that the diagnosis of SOP depending on the localization of fundus excyclotorsion might be misled. The results of our study solidify this previous notion. In this study, we demonstrated that 8.5% of unilateral SOP patients showed bilateral fundus excyclotorsion. We compared objective and subjective excyclotorsion in the unilateral SOP_BE_ group and bilateral SOP group, and there were no significant differences in the fundus torsional angles between the two groups. Furthermore, most cases of unilateral SOP_BE_ showed symmetric fundus torsion in both eyes. These results suggest that the presence or the pattern of bilateral fundus excyclotorsion is not a distinguishing sign for bilateral SOP. Instead, subjective excyclotorsion was significantly greater in bilateral SOP, and therefore should be considered an important factor for distinguishing bilateral and unilateral SOP. This finding is consistent with the previous observations in which subjective excyclotorsion, mostly measured by the double Maddox rod test, was significantly greater in bilateral SOP than in unilateral SOP [2,3,5,27,28]. One thing to consider when interpreting the result is that patients with unilateral SOP were mostly congenital, and patients with bilateral SOP were mostly acquired in this study. However, one patient with bilateral congenital SOP showed subjective excyclotorsion of 10 degrees, which is larger than most of the patients with unilateral congenital SOP with an average value of 4.6 degrees. Subjective excyclotorsion still seems to be useful in differentiating bilateral SOP from unilateral SOP, however, further investigations based on the etiology would be helpful. 

The contributing factors causing bilateral excyclotorsion in unilateral SOP remains to be elucidated. One possible contributing factor is ocular dominance and neural adaptation, which may partially explain bilateral excyclotorsion in unilateral SOP. According to Kim et al., ocular dominance and conjugate cycloversion movements might influence the torsional position of both eyes, and according to the amount of excyclotorsion, the paretic or non-paretic eye would be judged as normal or “extorted” [26]. It has also been known that the Listing’s plane, the plane of axes for all rotations that start from the primary position, are displaced in paralytic strabismus and have neural adaptability [29,30]. This neural adaptation occurs in a way that may allow the eye to regain efficiency in motor control and visual function [31]. Therefore, when the paretic eye is the sighting dominant eye, prolonged fixation of the paretic eye might reduce the amount of excyclotorsion in that eye, reversely inducing an excyclorotation of the non-paretic fellow eye by conjugate cycloversion movements according to Hering’s law. According to the amount of conjugate cycloversion movements, the paretic or non-paretic eye would be judged as normal or “extorted”. This also could partially explain the interesting finding that the majority of patients in the unilateral SOP_BE_ group showed symmetric bilateral excyclotorsion between the paretic eye and non-paretic eye. Whether bilateral excyclotorsion appears symmetrical or asymmetrical is thought to be the result of the amount of conjugate cycloversion movements [26]. Further studies regarding the contributing factors of bilateral excyclotorsion in unilateral SOP are necessary to clarify this issue.

Another possible explanation of unilateral SOP_BE_ may rely on anatomical pathologies. Interestingly, three patients in the unilateral SOP_BE_ group, including the representative case in Figure 1, were found with heterotopic rectus muscle pulleys in both orbits combined with unilateral SO hypoplasia. In these patients, rectus muscle pulleys in both orbits were displaced in the excyclotorsional direction. Previously, there have been studies reporting that rectus pulley displacements can be found in SO palsy and can simulate SO palsy [19,32]. Suh et al. have described rectus pulley displacement in unilateral SOP [33]. However, these finding differ from those in our study in that the rectus pulley displacement was observed only in the ipsilesional side of unilateral SOP; contralesional pulleys were normally located. The presence of bilateral rectus pulley displacement in unilateral SOP and its relationship with bilateral fundus excyclotorsion remains to be elucidated. Nevertheless, concomitant pulley heterotopy in unilateral SO hypoplasia may be considered as one of the causes of bilateral fundus excyclotorsion in unilateral SOP.

There are several limitations in the present study. First of all, this was a retrospective study and the sample size was small, especially in the bilateral SOP group, which caused a disproportion between the unilateral and bilateral SOP groups. However, as we included only subjects who showed ipsilateral hypoplasia of the SO muscle in MRI in the unilateral SOP group, the diagnosis of unilateral SOP was confirmed by imaging, which is the strength of the present study. Further analyses with more cases of bilateral SOP can compare unilateral and bilateral SOP of the same etiology and may assist in understanding the mechanism of fundus excyclotorsion in detail. Secondly, as we measured fundus excyclotorsion angles with fundus photography, there might be possible errors related to fixation direction and measurement errors. We tried to minimize errors by measuring the angle twice and averaging it. Thirdly, the absolute cut-off value of pathologic fundus excyclotorsion is not defined. For this reason, we did not use the conventional fovea-to-disc angle, but instead we used the foveal displacement angle from the horizontal axis at the bottom of the optic disc, which was described in a previous study [9]. Lastly, as mentioned above, other possible contributing factors of bilateral excyclotorsion such as ocular dominance and horizontal or vertical deviations were not included in this study.

In conclusion, the present study is the first to investigate the presence and patterns of bilateral fundus excyclotorsion in patients with unilateral SOP. Unilateral SOP with ipsilateral SO hypoplasia may also show symmetric bilateral fundus excyclotorsion as in bilateral SOP. Therefore, objective fundus excyclotorsion may not be regarded as a distinguishing sign of bilateral SOP and other clinical findings should be sought to diagnose masked bilateral SOP.

## Figures and Tables

**Table 1:** Clinical characteristics of subjects with bilateral fundus excyclotorsion in unilateral superior oblique palsy (SOP) and bilateral SOP.

	Unilateral SOP_BE_ (*n* = 18)	Bilateral SOP (*n* = 7)	*p*-Value
Age at onset of sign/symptoms (y)	22.6 ± 21.1 (0–57)	49.1 ± 11.4 (34–65)	0.005 ^d^
Symptom duration (y)	9.0 ± 12.6 (0.1–44.0)	0.7 ± 0.7 (0.2–2.0)	0.001 ^d^
Etiology			<0.001 ^e^
Congenital	18 (100%)	2 (28.6%)	
Acquired	0	5 (71.4%)	
Gender			0.856 ^e^
Male	7 (38.9%)	3 (42.9%)	
Female	11 (61.1%)	4 (57.1%)	
Stereoacuity (log arcsec)	3.5 ± 2.1 (1.4–3.5, *n* = 16)	2.5 ± 0.7 (1.8–3.5, *n* = 7)	0.089 ^d^
Laterality of SOP			
Right	10 (55.6%)	n/a	
Left	8 (44.4%)	n/a	
Fundus excyclotorsion angle ^a^ (°)			
IL	5.7 ± 4.7 (0.7–17.0)	7.6 ± 4.3 (4.1–15.7)	0.125 ^d^
CL	5.1 ± 2.7 (0.7–11.0)	7.1 ± 4.7 (3.0–16.4)	0.423 ^d^
Inter-eye excyclotorsion angle differences (IL-CL) ^b^	0.7 ± 3.6 (−4.6–9.2)	0.5 ± 5.8 (−5.3–12.4)	0.615 ^d^
Subjective excyclotorsion ^c^	(*n* = 14)	(*n* = 6)	
Angle	4.6 ± 4.3 (−5.0–10.0)	16.0 ± 5.5 (8.0–20.0)	0.002 ^d^
IL	12 (66.7%)	6 (100%)	
CL	1 (5.6%)	0 (0%)	

Data are mean ± standard deviation (range) or n (%). SOP, superior oblique muscle palsy; unilateral SOP_BE_, unilateral SOP with bilateral excyclotorsion; y, years; IL, ipsilateral (paretic eye of unilateral SOP, hypertropic eye in primary gaze of bilateral SOP); CL, contralateral (non-paretic eye of unilateral SOP, hypotropic eye in primary gaze of bilateral SOP); n/a, not applicable. ^a^ Degree of rotation on fundus photographs; ^b^ difference in the fundus excyclotorsion angle between both eyes; “paretic eye–non-paretic eye” in unilateral SOP, “hypertropic eye in primary gaze–fellow eye” in bilateral SOP; ^c^ subjective excyclotorsion ≥ 5° by the double Maddox rod test; ^d^ Mann–Whitney U test, unilateral SOP_BE_ group vs. bilateral SOP group; ^e^ Pearson’s Chi-square test, unilateral SOP_BE_ group vs. bilateral SOP group.
